# Fetal and maternal surveillance in high-risk pregnancy: tools, timing, and trends

**DOI:** 10.25122/jml-2025-0105

**Published:** 2025-08

**Authors:** Madalina Piron-Dumitrascu, Dragos Cretoiu, Valentin Nicolae Varlas, Nicolae Suciu

**Affiliations:** 1 Department of Obstetrics and Gynecology Polizu Clinical Hospital, Alessandrescu-Rusescu National Institute for Mother and Child Health, Bucharest, Romania; 2 Department of Obstetrics and Gynecology, Carol Davila University of Medicine and Pharmacy, Bucharest, Romania; 3 Department of Medical Genetics, Carol Davila University of Medicine and Pharmacy, Bucharest, Romania; 4 Fetal Medicine Excellence Research Center, Alessandrescu-Rusescu National Institute for Mother and Child Health, 020395, Bucharest, Romania; 5 Department of Obstetrics and Gynecology, Filantropia Clinical Hospital, Bucharest, Romania

**Keywords:** stillbirth, neonatal outcomes, high-risk pregnancy, diagnosis, reduced fetal movements, fetal and maternal surveillance

## Abstract

According to WHO statistics, stillbirths represent an incompletely elucidated, partially neglected problem, resulting in millions of pregnancies per year globally. This phenomenon has a major emotional impact on parents and society, as well as an additional economic effort on the part of health services. Stratification of high-risk pregnancies could be followed by a decrease in perinatal mortality through careful monitoring and possible obstetric interventions in selected cases. Identification of risk factors, assessment of genetic causes, planning of imaging monitoring strategy, cardiotocography, and therapeutic management can contribute to a decrease in the number of stillbirths. In this narrative review, we aimed to assess the current status of fetal and maternal surveillance in high-risk pregnancies and the role of identifying fetal movements associated with the risk of stillbirth. Recommendations for routine monitoring of fetal movement are warranted in high-risk pregnancies, particularly those with placental pathology or small for gestational age (SGA)/FGR (fetal growth restriction) assessed by ultrasound or by analysis of various biomarkers. Current methods for fetal movement counting do not demonstrate high sensitivity and specificity, underscoring the need for further research. Identifying the main risk factors for stillbirth and stratifying fetuses at high risk will contribute to improving mater-nal-fetal outcomes and better management of health system resources.

## INTRODUCTION

Monitoring fetal health during pregnancy is a challenge for both the pregnant woman and the public health services. Fetal movements experienced by pregnant women indicate that the fetus is developing in both size and strength. These initial movements are typically felt by the mother between the 16^th^ and 22^nd^ weeks of pregnancy [1]. Thus, the first body movements have been identified at a gestational age of 7 weeks and 2 days [2]. In recent years, home pregnancy monitoring has become increasingly well-established, being an important tool in the early detection of reduced fetal movements (RFM), and in the diagnosis of uncertain fetal status [3]. Reduced fetal movements are an important parameter of fetal well-being. In a cohort study involving 101,597 women between 2009 and 2019, 8.7% presented with RFM at least once, and the rate of stillbirths after 28 weeks of gestation was 2.0 per 1,000 births [4]. In 2015, there were an estimated 2.6 million stillbirths in the third trimester, most of which occurred in low- and middle-income countries [5]. In 2019, there were 2 million stillbirths at ≥28 weeks of gestation, with a global stillbirth rate of 13.9 per 1,000 births [6]. The target by 2030 is for the rate of stillbirths to be <12 per 1,000 live births, caused by impaired placental function, either with fetal growth restriction (FGR) or preterm labor [5].

Although the National Institute for Clinical Excellence (NICE) [7] advised in 2004 against routine fetal movement counting due to its limited impact on preventing fetal death, subsequent studies have supported its role in fetal monitoring [8,9]. In a prospective study, pregnant women who applied the 'count-to-ten' method from 34 weeks of gestation showed reduced delays in seeking care after reporting reduced fetal movements (RFM), which was associated with a lower stillbirth rate (3.06 per 1,000 births) [10]. Establishing the normal range of maternal perception time for counting fetal movements within 30 minutes, using the 'count-to-ten' method, will increase the perception of RFM, especially in the third trimester of pregnancy [11].

Pregnant women with FGR who experienced difficulties perceiving RFM presented less frequently to outpatient services than those without FGR, underscoring the need for careful monitoring in this high-risk group [12].

Assuming that 40% of stillbirths occur after 36 weeks of gestation in fetuses without structural abnormalities, Armstrong-Buisseret *et al*. in a multicenter, randomized, controlled pilot trial (ReMIT-2), compared usual care versus placental biomarker-based care in pregnant women with RFM after 36+ 0 weeks of gestation, to assess the association of abnormal placental function with poor neonatal out-comes [13].

In resource-limited settings, it has been observed that women are instinctively aware of fetal movements (FM), but they are often unable to monitor FM properly or determine when to report concerns about FM to healthcare providers. Therefore, both education and FM management protocols are needed. In a prospective cohort study of 305 women presenting with RFM after 28 weeks of gestation, 22.1% of pregnancies had a poor perinatal outcome (SGA), an outcome closely related to factors dependent on placental dysfunction. Therefore, new placental function tests are needed to better assess the associated fetal response [14]. Huang *et al*. conducted a study involving 1,147 pregnant women who used the Count the Kicks (CTK) mobile phone app to track their daily fetal movements. They found that pregnant women did not fully comply with the recommendation to monitor fetal movements in the last trimester of pregnancy, but had lower levels of anxiety [15].

Formal fetal movement counting during pregnancy does not negatively impact maternal psychological or emotional well-being and may enhance maternal-fetal attachment [16].

Providing pregnant women with information about the importance of counting and assessing fetal movements, identifying a normal pattern, and recognizing alarm elements can facilitate the establishment of an active therapeutic behavior [17]. In a retrospective cohort study of 591 women presenting with RFM after 24 weeks of gestation, the incidence of RFM was 22.6% (range, 14.9%-32.5%), with more than one presentation of RFM in 46.2% of cases. An increased number of visits was found, which led to an increase in resource use and obstetric interventions, without evidence of an increase in perinatal mortality, neonatal intensive care unit (NICU) admission, abnormal cardiotocography, or severe morbidity represented by Apgar score <7 at 5 minutes, arterial pH <7.0, or encephalopathy [18].

The identification and correct management of RFM are also important from the perspective of resource allocation by public health services. Thus, the AFFIRM study (a cluster-randomized, stepwise trial conducted in 33 hospitals in the UK and Ireland) showed an increase in direct costs of £95,126 per 1,000 births, and aimed to implement a care pathway to reduce stillbirths secondary to the identification of RFM. Although it represents a major public health problem in terms of cost-effectiveness, due to the low number of perinatal deaths, the implementation of this program needs to be re-evaluated in the context of the evolution of stillbirth rates [19].

Another risk is the timing of RFM presentation during pregnancy. The rate of stillbirths in prolonged pregnancies is approximately 14.0% [5]. Thus, in a prospective, single-center, randomized, open-label study (the COMPTAMAF study), in which 278 patients with prolonged pregnancies par-ticipated, no differences in neonatal morbidity rates were observed; fetal movement counting did not result in a reduction in adverse neonatal outcomes [20].

In this narrative review, we aimed to assess the current status of fetal and maternal surveillance in high-risk pregnancies and the role of identifying fetal movements associated with the risk of stillbirth.

## PREGNANCY SURVEILLANCE AND REDUCED FETAL MOVEMENTS – DIAGNOSIS AND THERAPEUTIC MANAGEMENT

Reduced fetal movements are a significant clinical concern during pregnancy, often associated with adverse perinatal outcomes, including FGR, preterm birth, and stillbirth. Early recognition and appropriate management are crucial for optimizing maternal and fetal outcomes. Maternal perception of fetal movement remains a key subjective indicator of fetal well-being, although it can vary significantly and does not always correlate with objective ultrasound findings [17].

Counting fetal movements poses challenges due to a lack of consensus on the best approach, as there is no universally accepted standard for diagnosing RFM. It is typically based on maternal self-report, often guided by midwives or obstetricians. Fetal movement assessment typically follows two main approaches:

- Fixed time method: Movements are counted over a defined time interval, traditionally 12 hours but often modified to shorter periods.
- Fixed number method: The time required to perceive a predetermined number of movements (commonly 10) is recorded. This method is generally associated with higher maternal com-pliance [21].

Despite their widespread use, evidence supporting these methods as reliable predictors of fetal compromise is limited. Maternal perception can be influenced by multiple factors, including anterior placenta, oligohydramnios/polyhydramnios, maternal body position, medication, stress, anxiety, and smoking, and can vary as pregnancy progresses [22].

Research has established a link between RFM and pregnancy complications, including preterm birth, FGR, and stillbirth [14]. Monitoring fetal movements is a common method used by both healthcare providers and expectant mothers to assess fetal health. Movement typically increases between weeks 16 and 36 of gestation, with a slight decline during the final month of pregnancy. Variations in fetal movements throughout pregnancy are normal and influenced by factors such as amniotic fluid level, fetal position, maternal medications, and fetal well-being. Ultrasound can categorize fetal activity into four types of movements: trunk (including hiccups, rotations, and breathing), limbs, face, and head. However, what a mother feels may differ from what is observed via ultrasound, with studies showing maternal detection of only 33–88% of the visible movements [23]. RFM is typically self-reported, guided by advice from midwives and obstetricians. Healthcare professionals need to educate pregnant women about the significance of being aware of fetal movements. However, encouraging self-monitoring may also heighten maternal anxiety [24].

Although the commonly described mechanism is reduced active fetal movement, 10–30% of women who will deliver a stillbirth have been identified with single episodes of excessive fetal movement preceding fetal death. These episodes may be associated with the presence of fetal convulsions due to fetal asphyxia or umbilical cord pathology (true cord knot) [25].

However, a systematic review concluded there is limited evidence supporting fetal movement counting as a reliable indicator of fetal health [26]. Current guidelines from the Royal College of Obstetricians and Gynaecologists stress that the mother’s subjective sense of reduced movement is what matters most [27].

Emerging strategies, such as the 'mindfetalness' approach that encourages women to consciously observe fetal movements for 15 minutes daily while lying on their left side, have been studied in a randomized trial performed by Akselsson *et al*. [28]. Although this method was associated with an increased number of RFM-related medical visits, it did not result in a reduction in the number of newborns with low Apgar scores. Interestingly, it was linked to a lower rate of cesarean sections and fewer cases of small for gestational age (SGA) infants [28].

The study by Malm *et al*. analyzed the effectiveness of two questionnaire-based methods of self-assessment of fetal movement monitoring in a group of 40 pregnant women: the 'count-to-ten' method and the 'mindfetalness' method. The final result was that the 'mindfetalness' method was preferred, being associated with an increased degree of relaxation and safety compared to the 'count-to-ten' method [29].

Antenatal care aims to identify pregnancies at risk and prevent negative outcomes for both mother and baby, while avoiding unnecessary medical intervention. Most pregnancies with RFM proceed normally, but some involve elevated risks. Since there is no standardized national protocol for managing RFM, clinical responses vary by region, often involving examinations or induced deliveries based on local guidelines and practices. The number of women seeking care for RFM has been on the rise.

A 2012 Cochrane review noted the absence of randomized controlled trials on RFM management and insufficient evidence to determine the most effective strategy [30]. Data from Norway indicate that ultrasound, in addition to cardiotocography (CTG), is crucial for evaluating fetal health, as it detects significant abnormalities in 11.6% of RFM cases, which often influence clinical decisions [9].

A cohort study of women undergoing planned cesarean sections found that fetuses with no observable movements on ultrasound just before delivery had lower umbilical cord pH, lower oxygen and base excess levels, and higher CO_2_ compared to those with active movements [31]. An international survey (the STARS cohort study) involving 1,714 women who experienced stillbirths after 28 weeks found that 30.5% noticed markedly reduced movements, 8.5% felt an increase, and 28% reported no change before the event [32].

RFM remains a vital clinical sign requiring thorough evaluation. In the absence of standardized diagnostic and therapeutic guidelines, a combination of maternal history, clinical judgment, and adjunct investigations (CTG, ultrasound) is essential. Increasing maternal awareness while avoiding unnecessary interventions remains the cornerstone of effective management. Further high-quality studies are needed to establish evidence-based protocols for RFM.

## STILLBIRTH – CLASSIFICATION, RISK FACTORS, DIAGNOSIS, AND RECOMMENDATIONS

Stillbirths continue to be a global concern despite concerted public health action. Thus, 2.6 million cases are recorded annually, of which approximately half are identified during pregnancy. According to the World Health Organization, 75% of stillbirths occur in South Asia and sub-Saharan Africa [33]. Population demographic studies have observed an increased rate of women of advanced reproductive age, higher rates of body mass index (BMI), and associated comorbidities.

To reduce the rate of stillbirths, a series of proactive clinical interventions is needed. In Norway, women who reported RFM were included in studies based on clinical protocols (non-stress tests, ultrasound assessment of fetal movements and growth, amniotic fluid volume) applied within 2 hours of the absence of fetal movements. This action resulted in a 1.8% reduction in stillbirths for RFM and a 1.0/1000 in general [9]. Another study (AFFIRM) conducted in the United Kingdom aimed to increase awareness of RFM and introduce standardized management. The conclusion of this study showed an insignificant reduction in stillbirths, but a significant increase in the rates of cesarean delivery and induction of labor [19].

The role of cardiotocography (CTG) assessment at admission and continuously during labor and delivery is important in reducing intrapartum stillbirths, compared to hospital care without CTG, which led to an 18-fold higher risk of stillbirths. Another prospective observational study conducted between 2008 and 2010, on a group of 160 patients, observed that CTGs on admission were reactive in 77% of patients, equivocal in 14.4%, and hypo-/areactive in 8.7% of women, which proves the importance of using this simple, non-invasive test in the triage of high-risk fetuses [34].

Gyllencreurz *et al*. studied abnormal CTG at admission in 127,461 low-risk pregnant women in Sweden and undetected SGA fetuses. They identified a rate of 4.9% of abnormal CTG with a proportion of 18.6% SGA fetuses, compared with a normal CTG group in which SGA fetuses were encountered with a frequency of 9.7%. The association of SGA with abnormal CTG at admission increased more than 20-fold the risk of severe adverse neonatal outcomes such as Apgar score less than 4 to 5 minutes, grade 2-3 hypoxic-ischemic encephalopathy, neonatal seizures, or even neonatal death [35].

Currently, the multitude of classification systems for stillbirths complicates the decision tree for determining the cause of death. These are based on fetal, maternal, or both factors, with significant differences in diagnosis reported between high-income countries and low-income countries. Strategies to reduce stillbirth rates include increasing rates of induction of labor and cesarean sections, reducing the gestational age at which induction is performed, and stratifying high-risk pregnancies [5].

In high-income countries, approximately 90% of stillbirths occur in the antepartum period, with the rate of third-trimester stillbirths ranging from 1.3 to 8.8 per 1000 births. This implies that public health efforts to reduce it may need to be intensified [36]. In the Nordic countries, early induction of labor did not significantly alter perinatal outcomes (stillbirth, neonatal death), but increased the risk of potential complications.

Identification of high-risk pregnancies with small-for-gestational-age infants in Sweden showed a 40% rate of stillbirths < 37 weeks and 11% > 37 weeks for these infants. Another effective direction that could be pursued would be targeted ultrasound screening of high-risk groups in the third trimester of pregnancy. Thus, routine ultrasound assessment at 32 weeks of gestation of SGA infants observed a four-fold reduction in the number of stillbirths and neonatal deaths [37]. A Cochrane review reported a decrease in the risk of stillbirths of approximately 20-fold in those examined late by ultrasound [38].

## RISK FACTOR ASSESSMENT AND MANAGEMENT

### Placental abnormalities and Doppler evaluation

Since the placenta is an organ with a crucial role in fetal growth and development, dysfunctions in gas exchange, nutrient transfer, hormone secretion, and immune status can cause serious complications in pregnancy and the neonatal period (SGA, preeclampsia, and stillbirth). Thus, pathological examination of the placenta is mandatory in cases of stillbirth. Placental studies have identified structural and functional abnormalities (small, hypovascularized, with areas of infarction, with villous hypovascularization and diminished trophoblastic function, with signs of endocrine dysfunction) [39].

Placental dysfunctions are associated with compensatory hemodynamic changes in the fetus, resulting in a redistribution of blood flow to the brain ('brain preservation' effect) and other essential organs, a phenomenon frequently observed in SGA fetuses with adverse perinatal and long-term neurodevelopmental outcomes [40]. The degree of placental damage can be assessed using the cerebroplacental ratio (CPR). Doppler examination of the uterine arteries is a non-invasive method for assessing placental function, with clinical applicability in both the first and third trimesters of pregnancy [41].

The study by Prior *et al*. showed that measurement of the cerebroplacental ratio 72 hours ante-natally can identify fetuses with an increased potential for intrapartum compromise and requiring ob-stetric interventions [42]. Triunfo *et al*. reported that Doppler assessment of fetal vessels and maternal uterine artery at 37 weeks in a group of low-risk pregnancies improved the prediction of adverse perinatal outcomes [43]. Another retrospective cohort study of 2,812 patients showed that CPR assessed in the third trimester is an independent predictor of stillbirth and perinatal mortality in a mixed-risk population [41].

Rial-Castelo *et al*. in 1,030 low-risk pregnancies examined by Doppler at 32–34 weeks did not find any improved predictive value of CPR and uterine Doppler over standard screening practice for impaired fetal growth [40].

In another study of low-risk women at term, uterine artery pulsatility index (PI) values >95^th^ percentile and CPR values <10^th^ percentile were observed in cases requiring cesarean section for intrapartum fetal compromise and in those with neonatal morbidity [44]. CPR at 35–37 weeks of gestation is a poor predictor of adverse perinatal outcomes [45], and CPR below the 5^th^ percentile was significantly associated with an increased risk of perinatal mortality [46]. Similarly, a study by Bligh *et al*. in low-risk women beyond 36 weeks found no significant association between the placental growth factor (PlGF) blood test and CPR in predicting cesarean delivery for intrapartum fetal compromise [47].

Choorakuttil *et al*., in a group of 1,326 pregnant women in the third trimester of pregnancy, revealed 308 (23.23%) cases with abnormal Doppler values, 11 (0.83%) cases of stillbirths, and 11 (0.84%) neonatal deaths. Thus, Doppler evaluation was important for identifying late stillbirth, but not for term stillbirth or neonatal deaths [48]. In the last trimester of pregnancy, an elevated PI value on the uterine artery is rare; cerebral blood flow redistribution and low CPR are associated with an increased risk of perinatal complications and stillbirth [49].

### Angiogenic/antiangiogenic factors

The process of angiogenesis can be influenced multifactorially, supporting tissues through oxygen and nutrient supply, waste elimination, and immune response. It is regulated by pro-angiogenic and antiangiogenic factors, which are essential for biological processes such as reproduction and wound healing. Thus, vascular endothelial growth factor (VEGF), especially VEGF-A, is the central regulator of both angiogenesis and vasculogenesis [50,51].

The stimulation of angiogenic molecule production (VEGF) in endothelial cells under low oxygen conditions occurs through HIFs (hypoxia-inducible factors). As a result, the vascular function of VEGF occurs through its receptors, mainly VEGFR-1 (sFlt-1) and VEGFR-2 (KDR/Flk-1) [52].

Other growth factors involved are: platelet-derived growth factor (PDGF), transforming growth factor beta (TGF-β), and angiopoietins (Ang-1 and Ang-2). Endoglin, a co-receptor of the TGF-β family, is also upregulated in hypoxia and acts alongside VEGF to promote angiogenesis, compared to soluble endoglin (sEng), which acts as an inhibitor of angiogenesis. Soluble tyrosine kinase-1 (sFlt-1) binds to VEGF and PlGF, preventing their interaction with endothelial receptors in the placenta and thereby inhibiting angiogenesis [53].

Normal placental function is defined by a balance between pro- and antiangiogenic factors, and any dysfunction in tissue oxygen delivery can cause conditions such as preeclampsia, FGR, and gestational hypertension [54]. Nanjo *et al*. found that levels of circulating angiogenic and antiangiogenic factors near delivery were correlated with the severity of hypertensive disorders and FGR [55].

Elevated levels of HIF-1 and HIF-2 have been observed in the placenta in cases of chronic hypoxia, such as preeclampsia and FGR. Pregnancies that end in stillbirth often have altered angiogenic profiles (low PlGF and high sEng and sVEGFR-1) [56].

Chaiworapongsa *et al*. demonstrated that maternal blood testing between 24 and 28 weeks of gestation for PlGF, PlGF/sVEGFR-1, and PlGF/sEng ratios can predict fetal death. When these values fell below the 2.5^th^ percentile, there was a 29-fold increased risk of stillbirth, with a false-positive rate of only 3.5% [57]. Elevated maternal sVEGFR-1 levels may reflect the presence of an antiangiogenic state that may explain some stillbirths [58].

### Microbiome

In recent years, research on the gut-brain-microbiome axis has highlighted the bidirectional communication between the central nervous system and the gastrointestinal tract, a connection that represents a key point in the study of health and disease. Furthermore, microbiota dysfunction can be associated with the disruption of physiological processes in multiple systems. Increasing evidence suggests that microbial populations in the gut, vagina, and other sites are relevant during pregnancy, potentially influencing the risks of preterm birth, preeclampsia, gestational diabetes, and excessive maternal weight gain [59].

Molecular methods and next-generation sequencing have suggested the possible presence of a placental or uterine microbiome that may play a role in early fetal colonization [60]. However, in a comprehensive review, Perez-Muñoz *et al*. reaffirmed support for the concept of a sterile uterus, citing issues of contamination and unverifiable microbial identification in many studies [61].

More recent research, including a study by Parnell *et al*., has challenged this claim by identifying unique microbial profiles in different regions of the placenta, regardless of the method of delivery [62]. In particular, the presence of bacteria in amniotic fluid or the placenta has been associated with adverse outcomes, such as spontaneous abortion, preterm labor, SGA, neonatal sepsis, postpartum infections, and stillbirth.

### Environmental factors

Stillbirths frequently occur through causal pathways involving impaired placental function, such as fetal growth restriction or premature birth. Some persistent organic pollutants have endocrine-disrupting effects due to their potential to adversely affect pregnancy outcomes. Thus, Roncati *et al*. demonstrated the presence of organochlorine pesticides used in apple cultivation in the brains of 11 out of 24 stillborn fetuses. These pesticides crossed the placental barrier by passive diffusion, even at low-dose exposure, and can penetrate the fetal blood-brain barrier [63].

Prenatal exposure to adverse environmental conditions, such as high ambient temperatures and air pollution, is associated with an increased risk of stillbirth. Air pollution, defined as ambient concentrations of fine particulate matter (PM2.5) ≤2.5 μm, tends to be higher in socioeconomically disadvantaged areas, with a mean concentration of 1.13 μg/m^3^ in vulnerable regions [64].

Ambient temperature fluctuations have been associated with an increased risk of stillbirth, particularly among women of advanced maternal age and lower socioeconomic status. A 10°F rise in ambient temperature during the week preceding delivery was linked to a 45% higher risk of stillbirth [65]. Similarly, an increase of 1°C (1.8°F) during the week prior to delivery was associated with a 6–7% increase in the risk of fetal death [66,67].

### Other factors

The proteomic profile of plasma extracellular vesicles (EVs) from pregnant women with stillbirth revealed the presence of 19 proteins including placental growth factor, macrophage migration inhibitory factor, endoglin, RANTES, interleukin (IL)-6, IL-8, IL-16, macrophage inflammatory protein-1α, urokinase plasminogen activator surface receptor, tissue factor pathway inhibitor, E-selectin, vascular endothelial growth factor receptor-2, pentraxin 3, galectin-1, monocyte chemotactic protein-1, disintegrin and metalloproteinase domain-containing protein 12, insulin-like growth factor-binding protein-1, matrix metalloproteinase-1 (MMP-1), and CD163. Thus, three distinct groups of stillbirth cases with varying clinical and placental histological manifestations were identified, resulting from the combination of plasma concentrations of EV and soluble proteins [68]. The study of beat-to-beat heart rate, based on electrophysiology and fetal movement index using fetal magnetocardiography, identified that maternal metabolic control in gestational diabetes may influence the regulation of fetal autonomic heart rate by increasing fetal vagal tone, without affecting sympathetic tone and motor activity [69].

## DISCUSSION

The presence of normal fetal movements at various stages of pregnancy is a crucial component of central nervous system integrity and fetal well-being. Alterations in movement patterns, as detected by counting techniques, may signal reduced oxygenation and progression toward fetal compromise. This monitoring of fetal movements can be valuable in high-risk pregnancies, but also routinely, with the mention that excessive use of these techniques may increase the level of anxiety of pregnant women [26].

According to current clinical guidelines, the risk of stillbirth can only be determined to a limited extent based on the identification of reduced fetal movements. Thus, fetal assessment, which involves corroborating the information obtained from Doppler ultrasound and cardiotocography, relates to the current fetal status and, to a small extent, to its predictability. The role of this assessment increases in the case of monitoring a high-risk pregnancy. As a result, the frequency of examination of these preg-nancies that may become complicated and the choice of the optimal time for resolution continue to represent real clinical challenges, and additional randomized clinical trials are needed to elucidate this issue.

In women with pregestational diabetes compared with non-diabetics, there was a 4-fold higher rate of stillbirths, 83% of which were without congenital malformations [70]. Freeman *et al*. showed an antepartum fetal death rate in the non-stress test group eight times higher than in the group of pregnant women with contraction stress test (3.2/1,000 versus 0.4/1,000) [71]. In a prospective, multicenter cohort study of 7,934 women with singleton births at or after 24 weeks of gestation, a low level of PAPP-A collected at 10 weeks of gestation was associated with stillbirth secondary to placental pathology (placental abruption or unexplained stillbirth associated with FGR) [72].

Kniffka *et al*. evaluated stillbirths from 2010 to 2021 in 25 European countries and found an increase correlated with older maternal age and a decrease secondary to a reduction in multiple pregnancies [73].

The use of fetal kick charts as a predictor of stillbirth rates remains controversial, with some studies documenting this particularly in high-risk pregnancies, while other observational studies have shown no change [74]. In a retrospective cohort study of women who experienced reduced fetal movement after 22 weeks of gestation, an increased risk of adverse neonatal outcome (18.4%) was found in SGA pregnancies and (12.8%) in large-for-gestational-age (LGA) fetuses obtained by in vitro fertilization [75].

Assessment of fetal well-being and developmental status can be achieved by analyzing the pattern of fetal movements. Home monitoring of high-risk pregnancies using non-invasive fetal movement detection devices is challenging due to the low-amplitude waves that are strongly disturbed by background factors, necessitating the use of a Bayesian algorithm to optimize the fetal movement signal [76]. The development of advanced fetal movement monitoring systems based on artificial intelligence can enhance the accuracy of fetal movement detection and the degree of prediction, enabling early inter-vention and a reduction in the fetal death rate. Another study observed that digital signal processing increased the accuracy of the device, and coupling to GSM (Global System for Mobile Communications) allowed remote monitoring of pregnant women with high obstetric risk [77].

In long-term fetal monitoring, portable triaxial accelerometers placed on the maternal abdomen are used to differentiate true fetal movements from artifacts, providing objective data on movement characteristics [78]. In a meta-analysis based on data from several maternity units in the United Kingdom, in which 1,175 pregnant women with RFM between 28+0 and 41+0 weeks of gestation participated, adverse pregnancy outcomes (abnormal fetal heart rate, smoking, maternal medical history) were found in 7.7% of pregnancies with RFM [79].

Identification of the main risk factors for stillbirth (e.g., advanced maternal age, infectious processes, preeclampsia, gestational diabetes, noncommunicable diseases, nutritional status, obesity, smoking, inadequate pregnancy monitoring, FGR/SGA, preterm labor, prolonged pregnancy, inadequate medical infrastructure) contributes to a significant reduction in the rate of unavoidable stillbirths. For example, the rate of stillbirths after 28 weeks of gestation caused by congenital anomalies represents only 7.4% of them, while over 50% occur during labor [5]. Therefore, active intervention on these risk factors will contribute to improving maternal-fetal outcomes.

Despite technological advances, consensus on the optimal method of fetal movement monitoring remains elusive, particularly in low-resource settings. Emerging tools such as AI-assisted monitoring and biomarker screening hold potential but require further validation through large-scale clinical trials.

## CONCLUSION

Recommendations for routine monitoring of fetal movement are warranted in high-risk pregnancies, particularly those with placental pathology or SGA/FGR assessed by ultrasound or by analysis of various biomarkers. Current methods for fetal movement counting do not demonstrate high sensitivity and specificity, indicating the need for further research. Identifying the main risk factors for stillbirth, stratifying fetuses at high risk will contribute to improving maternal-fetal outcomes and better management of health system resources.
